# Supplementary material for: Honey bees bred for Varroa sensitive hygiene trait demonstrate resistance to chalkbrood disease
Source: PLoS One. 2025 Aug 27;20(8):e0329739. doi: 10.1371/journal.pone.0329739 (PMC12385354; doi:10.1371/journal.pone.0329739)
Supplement: S1 Table — (DOCX) [file pone.0329739.s001.docx]

**S1 Table. Summary of methods used in 2023 and 2024 and between locations.**

| **Method** | **2023 Minnesota** | **2024 Minnesota** | **2024 Baton Rouge** |
| --- | --- | --- | --- |
| Colony configuration | Two deep boxes | One deep box (additional boxes over queen excluder) | One deep box |
| Colony assessments prior to challenge | frames of bees  frames of brood | frames of bees  frames of brood | Not sampled, colonies standardized based on size at start |
| Chalkbrood challenge | Pollen patties | Spore spray solution | Spore spray solution |
| Quantification of chalkbrood (days post-challenge) | 7 days  14 days | 2 days  4 days  7 days  14 days | 2 days  4 days  7 days |
| Chalkbrood signs recorded | Mummies only | Early signs and mummies | Early signs and mummies |
| Freeze-killed brood assay | Pre-challenge | Pre-challenge | Pre-challenge |
| *Varroa* mite sample collection | Pre-challenge and end of season | Pre-challenge and end of season | Pre-challenge |
