# Supplementary material for: Honey bees bred for Varroa sensitive hygiene trait demonstrate resistance to chalkbrood disease
Source: PLoS One. 2025 Aug 27;20(8):e0329739. doi: 10.1371/journal.pone.0329739 (PMC12385354; doi:10.1371/journal.pone.0329739)
Supplement: S2 Table — Number of pooled pre-pupae were restricted by how many symptomatic pre-pupae were found on the comb. In the of colony 24429, only 6 pre-pupae were amorphous and only 14 pre-pupae were acuminate within the comb. (DOCX) [file pone.0329739.s002.docx]

**S2 Table. Sample sizes of pre-pupae pooled for detection of chalkbrood by RT-qPCR.**

| **Colony** | **Early sign seen** | **Number of pre-pupae pooled** |
| --- | --- | --- |
| 22130 | Acuminate | 20 |
| 22130 | Amorphous | 20 |
| 22130 | Re-Capped | 20 |
| 24429 | Acuminate | 14 |
| 24429 | Amorphous | 6 |
| 24429 | Re-Capped | 20 |
