# Supplementary material for: Honey bees bred for Varroa sensitive hygiene trait demonstrate resistance to chalkbrood disease
Source: PLoS One. 2025 Aug 27;20(8):e0329739. doi: 10.1371/journal.pone.0329739 (PMC12385354; doi:10.1371/journal.pone.0329739)
Supplement: S3 Table — (DOCX) [file pone.0329739.s003.docx]

**S3 Table. Primer sequences utilized for screening of pathogens by RT-qPCR**

| **Pathogen** | **Primer Sequence** | **Reference** |
| --- | --- | --- |
| Sacbrood | F: AACGTCCACTACACCGAAATGTC  R: ACACTGCGCGTCTAACATTCC | [1] |
| Chalkbrood | F: TCTGGCGGCCGGTTAAAGGCTTC  R: GTTTCAAGACGGGCCACAAAC | [2] |

**Bibliography**

1. Blanchard P, Guillot S, Antùnez K, Köglberger H, Kryger P, de Miranda JR, et al. Development and validation of a real-time two-step RT-qPCR TaqMan® assay for quantitation of Sacbrood virus (SBV) and its application to a field survey of symptomatic honey bee colonies. Journal of Virological Methods. 2014 Mar 1;197:7–13.

2. Evans JD. Beepath: An ordered quantitative-PCR array for exploring honey bee immunity and disease. Journal of Invertebrate Pathology. 2006 Oct 1;93(2):135–9.
