# Supplementary material for: Honey bees bred for Varroa sensitive hygiene trait demonstrate resistance to chalkbrood disease
Source: PLoS One. 2025 Aug 27;20(8):e0329739. doi: 10.1371/journal.pone.0329739 (PMC12385354; doi:10.1371/journal.pone.0329739)
Supplement: S4 Table — Binomial regression statistical results comparing chalkbrood mummy prevalence and early sign prevalence by queen type (POL-line vs. Commercial). At 4 days post challenge in Baton Rouge, Pol-line colonies had significantly (86.4%) lower odds of having chalkbrood mummies compared to Commercial colonies (β = −1.99, p = 0.034). wk = week, d = day. (DOCX) [file pone.0329739.s004.docx]

**S4 Table. Prevalence of chalkbrood mummies and early signs by queen type.**

| **Location & Year** | **Days post challenge** | **Prevalence of mummies by queen** | | | | **Prevalence of early signs by queen** | | | |
| --- | --- | --- | --- | --- | --- | --- | --- | --- | --- |
|  |  | β | SE | z | p | β | SE | z | p |
| Minnesota 2023 | 7 d | -5.9e-16 | 0.86 | 0 | 1 | – | – | – | – |
|  | 14 d | 0.25 | 0.96 | 0.26 | 0.79 | – | – | – | – |
| Minnesota 2024 | 2 d | – | – | – | – | 0.19 | 0.24 | 0.8 | 0.43 |
|  | 4 d | -0.28 | 0.99 | -2.9 | 0.77 | -0.09 | 0.19 | -0.49 | 0.63 |
|  | 7 d | -1.2 | 1.28 | -0.97 | 0.33 | 0.01 | 0.16 | 0.08 | 0.93 |
|  | 14 d | 1.28 | 1.30 | 0.98 | 0.32 | 0.32 | 0.26 | 1.25 | 0.23 |
| Baton Rouge 2024 | 2 d | 0.08 | -1.47 | 0.54 | 0.95 | 0.69 | 1.29 | 0.54 | 0.59 |
|  | 4 d | -1.99 | 0.94 | -2.12 | 0.03* | -18.86 | 4738 | -0.004 | 0.99 |
|  | 7 d | -18 | 4917 | -0.004 | 0.99 | 0.75 | 0.78 | 0.96 | 0.33 |
