# Supplementary material for: Honey bees bred for Varroa sensitive hygiene trait demonstrate resistance to chalkbrood disease
Source: PLoS One. 2025 Aug 27;20(8):e0329739. doi: 10.1371/journal.pone.0329739 (PMC12385354; doi:10.1371/journal.pone.0329739)
Supplement: S5 Table — Mean frames (fr) of bees and brood for Pol-line (Pol) and Commercial (Comm) colonies. Wilcoxon tests demonstrate difference between Pol-line and Commercial colonies. In 2023: Pol-line had significantly fewer frames of bees at day 0 (p = 0.03), however, there was no difference in frames of brood or brood pattern. In 2024: Wilcoxon tests revealed no statistically significant differences between Pol-line and Commercial colonies. (DOCX) [file pone.0329739.s005.docx]

| **Year** | **Pol mean fr bees ± sd** | **Pol mean fr brood ± sd** | **Comm mean fr bees ± sd** | **Comm mean fr brood ± sd** | **Pol vs. Comm fr bees** | | **Pol vs. Comm fr brood** | | **Pol vs. Comm br pattern** | |
| --- | --- | --- | --- | --- | --- | --- | --- | --- | --- | --- |
|  |  |  |  |  | W | p | W | p | W | p |
| 2023 | 12.8 ± 3.07 | 8.5 ± 2.5 | 15.2 ± 3.68 | 9.5 ± 2.3 | 92.5 | 0.03* | 76 | 0.30 | 71.5 | 0.44 |
| 2024 | 6.82 ± 1.1 | 5.89 ± 0.74 | 6.5 ± 1.1 | 5.6 ± 1 | 54 | 0.58 | 55 | 0.62 | 60 | 0.87 |

**S5 Table. Colony strength in 2023 and 2024 prior to challenge.**
