# Supplementary material for: Honey bees bred for Varroa sensitive hygiene trait demonstrate resistance to chalkbrood disease
Source: PLoS One. 2025 Aug 27;20(8):e0329739. doi: 10.1371/journal.pone.0329739 (PMC12385354; doi:10.1371/journal.pone.0329739)
Supplement: S6 Table — To investigate the relationship between Freeze Killed Brood (FKB) as a predictor of chalkbrood prevalence, a binomial regression model was used. A Firth Penalized Regression model was used if there was perfect separation of the variables. FKB and chalkbrood severity were analyzed via a linear model or Spearman Rank Correlation if the data was non-normal. Wk = week. d = day. FKB = Freeze-killed brood. (DOCX) [file pone.0329739.s006.docx]

**S6 Table. Hygienic behavior did not predict chalkbrood prevalence or severity.**

| **Location and Year** | **Days post challenge** | **FKB as a predictor of chalkbrood prevalence** | | | | | | **FKB as a predictor of chalkbrood severity** | | | | | |
| --- | --- | --- | --- | --- | --- | --- | --- | --- | --- | --- | --- | --- | --- |
|  |  | β | b | χ²(1) | SE | z | p | β | F | df | ρ | p |  |
| Minnesota 2023 | 1 wk | -0.002 | — | — | 0.05 | -0.46 | 0.65 | — | — | — | -0.01 | 0.95 |  |
|  | 2 wk | -0.006 | — | — | 0.004 | -1.37 | 0.18 | — | — | — | -0.19 | 0.29 |  |
| Minnesota 2024 | 2 d |  | — | — | — | — | — | — | — | — | — | — |  |
|  | 4 d | -0.005 | — | — | 0.006 | -0.85 | 0.65 | -0.005 | 0.73 | 15 | — | 0.40 |  |
|  | 1 wk | — | — | — | — | — | — | — | — | — | 0.05 | 0.84 |  |
|  | 2 wk | — | — | — | — | — | — | — | — | — | -0.01 | 0.96 |  |
| Baton Rouge 2024 | 2 d | 0.04 | — | — | 0.08 | 0.52 | 0.60 | — | — | — | -0.02 | 0.91 |  |
|  | 4 d | -0.01 | — | — | 0.02 | -0.46 | 0.65 | — | — | — | -0.14 | 0.49 |  |
|  | 1 wk | — | -0.002 | 0.003 | 0.042 | — | 0.95 | — | — | — | 0.14 | 0.47 |  |
