# Supplementary material for: Honey bees bred for Varroa sensitive hygiene trait demonstrate resistance to chalkbrood disease
Source: PLoS One. 2025 Aug 27;20(8):e0329739. doi: 10.1371/journal.pone.0329739 (PMC12385354; doi:10.1371/journal.pone.0329739)
Supplement: S7 Table — Spearman rank correlations indicated that FKB is not a predictor of mites/100 adult bees. FKB = Freeze-killed brood. (DOCX) [file pone.0329739.s007.docx]

| **Location and Year** | **FKB on June mite infestation** | | **FKB on September mite infestation** | |
| --- | --- | --- | --- | --- |
|  | ρ | p | ρ | p |
| Minnesota 2023 | -0.12 | 0.62 | -0.21 | 0.49 |
| Minnesota 2024 | 0.07 | 0.74 | 0.07 | 0.81 |
| Baton Rouge 2024 | -0.17 | 0.37 | — | — |

**S7 Table. Freeze-killed brood as a predictor of mite infestation.**
